# Microwave Imaging Sensor Using Low Profile Modified Stacked Type Planar Inverted F Antenna

**DOI:** 10.3390/s18092949

**Published:** 2018-09-05

**Authors:** Mohammad Tariqul Islam, Md. Amanath Ullah, Touhidul Alam, Mandeep Jit Singh, Mengu Cho

**Affiliations:** 1 Centre of Advanced Electronic and Communication Engineering, Faculty of Engineering and Built Environment, Universiti Kebangsaan Malaysia, Bangi, Selangor 43600, Malaysia; touhid13@siswa.ukm.edu.my (T.A.); mandeep@ukm.edu.my (M.J.S.); 2 Laboratory of Spacecraft Environment Interaction Engineering (LaSEINE), Kyushu Institute of Technology, Fukuoka 804-8550, Japan; cho@ele.kyutech.ac.jp

**Keywords:** PIFA, antenna, sensor, microwave imaging, SAR, unidirectional, design

## Abstract

Microwave imaging is the technique to identify hidden objects from structures using electromagnetic waves that can be applied in medical diagnosis. The change of dielectric property can be detected using microwave antenna sensor, which can lead to localization of abnormality in the human body. This paper presents a stacked type modified Planar Inverted F Antenna (PIFA) as microwave imaging sensor. Design and performance analysis of the sensor antenna along with computational and experimental analysis to identify concealed object has been investigated in this study. The dimension of the modified PIFA radiating patch is 40 × 20 × 10 mm^3^. The reflector walls used, are 45 mm in length and 0.2-mm-thick inexpensive copper sheet is considered for the simulation and fabrication which addresses the problems of high expenses in conventional patch antenna. The proposed antenna sensor operates at 1.55–1.68 GHz where the maximum realized gain is 4.5 dB with consistent unidirectional radiation characteristics. The proposed sensor antenna is used to identify tumor in a computational human tissue phantom based on reflection and transmission coefficient. Finally, an experiment has been performed to verify the antenna’s potentiality of detecting abnormality in realistic breast phantom.

## 1. Introduction

Medical imaging is one of the most common techniques for biomedical diagnosis to identify tumor or abnormality in the human body. Recently, the excessive use of some imaging modalities are raising issues regarding exposure to radiation and healthcare costs [1]. Radiation from conventional medical imaging system has become a critical health concern due to excessive radiation exposure [2]. Overuse of medical imaging-based diagnosis has brought increased exposure to ionizing radiation along with the increased danger of unwanted effects on human body [3]. Significant number of cancer deaths and cancer caused by medical imaging process that uses ionizing radiation, reported every year in the U.S. [4]. Researchers are trying to find alternatives to conventional diagnosis systems such as magnetic resonance imaging, computed tomography scan and other methods because they suffer from false-positive accuracy rate. Moreover, these processes are time-consuming and cannot facilitate portability [5] and most importantly, health concerns due to ionized exposure. For decades, these medical imaging systems have been used to detect any abnormality in the human body. Recently, the adverse impact on the human body and reliability issues raised by conventional imaging tools are being taken seriously [5].

Healthcare diagnosis tools based on microwaves are being given priority by researchers. Microwave signal contrast between the electrical characteristics of human tissues can easily be distinguished by microwave antenna sensors. Radiated and Scattered power are received by one or more antenna sensors in microwave imaging. Microwave-based portable medical diagnosis tools have the potential to save lives by utilizing microwave sensor antennas that perform well.

The concept of abnormality detection in the human body using microwave imaging sensors is based on the variation of contrast in dielectric properties in malignant and healthy tissue [6]. The water content of every biological tissue has different electrical properties. Change in dielectric properties can easily be sensed by microwave sensing antennas. Researchers specified some antenna properties as requirements for microwave imaging—unidirectional radiation pattern, a frequency range within 1–4 GHz and small dimensions to make the system portable are the most desired specifications [5,7].

Researchers are currently working to achieve all the antenna requirements for microwave imaging. Different types of microwave antennas have been reported for microwave-based biomedical diagnosis. They are mostly reported for human-head imaging and breast-cancer imaging [7,8,9,10,11]. In References [12,13], two antennas are presented for a heart failure detection system. In Reference [14], a folded and meandered loop antenna with three-dimensional structure was presented for medical diagnosis. Moreover, it was also proposed for the application of radio-frequency identification, ultrahigh frequency (UHF) TV channels and others. The antenna in Reference [10] achieved good operating bandwidth where the presented antenna in Reference [5] has low gain. Although, when antenna operates in lower microwave frequency good signal penetration can be achieved [14,15] but operating frequency in less than 1 GHz might blur the result of imaging [16]. A unidirectional antenna for microwave application was presented in Reference [17], that operates at 1.70–1.77 GHz with a pick gain of 3.42 dB. In Reference [7] the presented antenna covers good operating band. However, it is low in gain and the radiation pattern is not unidirectional which are key requirements. Moreover, the design is expensive substrate based where expenses in microwave imaging system in biomedical diagnosis is an issue as other costly imaging modalities are out of reach of large masses in developing countries [18]. A lens-loaded Vivaldi antenna has been introduced in Reference [19], which operates in ultra-wide band but it is large in dimension, gain is low at a lower microwave frequency with omnidirectional radiation. A unidirectional Vivaldi antenna has been introduced in Reference [20]. Though these antenna can cover a good operating band, the height of the Vivaldi antenna are usually high enough that it could be an issue while placement of imaging systems and substrate is also expensive. An interesting design of a dipole antenna was presented in Reference [21] for microwave imaging. However, the antenna is too high, the design is complex and the radiation pattern is not unidirectional, which is highly desired for microwave imaging. Though the peak gain of the antenna is 6.2 dB at 4 GHz but the gain is less than 3 dB at lower frequency. Another important factor in terms of better signal penetration in microwave imaging is high directivity. An antenna’s unidirectional characteristics can be validated by a high Front-to-back ratio, where the presented antenna in Reference [7] obtained a front to back ratio value of 9.

In this paper, a modified stacked type PIFA antenna as a microwave imaging sensor with a folded radiating structure for biomedical diagnosis is introduced. The significance of the proposed antenna are that it uses a modified stacked shape that can use full volume for impedance matching, providing a good effective electrical length for achieving resonance at a lower microwave frequency with high gain and the design facilitates an easier fabrication process for mass production (Stamping metal sheet method/3D printing) with an extremely low cost because the used copper material is very inexpensive. The proposed antenna achieved a maximum realized gain of 4.5-dB with a front-to-back ratio value of 15. Initially, the proposed antenna sensor was designed and analyzed in Computer Simulation Technology (CST) Microwave Studio. Then, it is manually fabricated using inexpensive copper sheet (0.2-mm-thick). The reflection coefficient performance of the fabricated prototype was tested using a PNA network analyzer. Finally, a computational sensitivity test of the proposed antenna as microwave imaging sensor has been carried out. Initially, the sensitivity test was performed using a computational tissue phantom based on a reflection and transmission coefficient. After that, a computation microwave imaging scenario has been performed to detect tumors in a three-layered rectangular human tissue phantom using the proposed microwave sensor antenna.

## 2. Design and Methodology

The proposed modified PIFA antenna has certain advantages over two-dimensional antennas.

**Figure 1:**
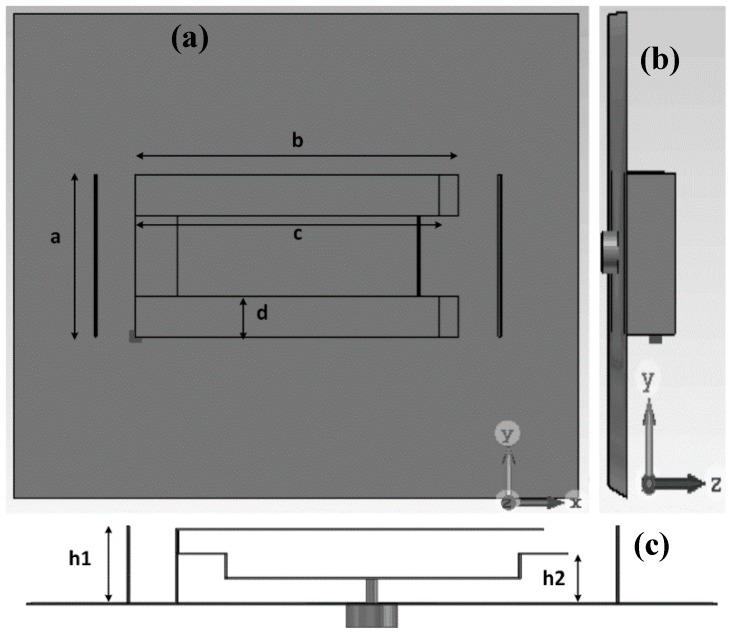
(**a**) Top; (**b**) side and (**c**) front views of the antenna.

The design can efficiently utilize full volume by stacking technique that can provide good effective length. Moreover, the fabrication process can be easier for mass production, using the stamping metal-sheet or three-dimensional (3D) printing technology. Figure 1 shows the design configurations of the antenna. The antenna configuration has been presented in different view for better understanding. The value of the proposed design parameters of the antenna are L = 70 mm, W = 60 mm, a = 20 mm, b = 40, c = 36 mm, d = 5 mm, h1 = 10 mm and h = 27.5 mm.

The aforementioned parameters are distinctively studied keeping the others fixed to investigate how the dimensional parameters influence the operating frequency of the proposed antenna. The folding technique is implemented for size reduction and getting a maximum effective electrical length. Moreover, the occurrence of phase alternation due to the folding enhances the directivity. We can see that the main radiating structure of the antenna can be realized using the folding technique, as shown in Figure 1c. To elevate the unidirectional property of the antenna, two reflector walls at both sides are used. To decrease the effect of spurious radiation that occurs in microstrip feeding, coaxial feeding is applied to the antenna.

**Figure 2:**
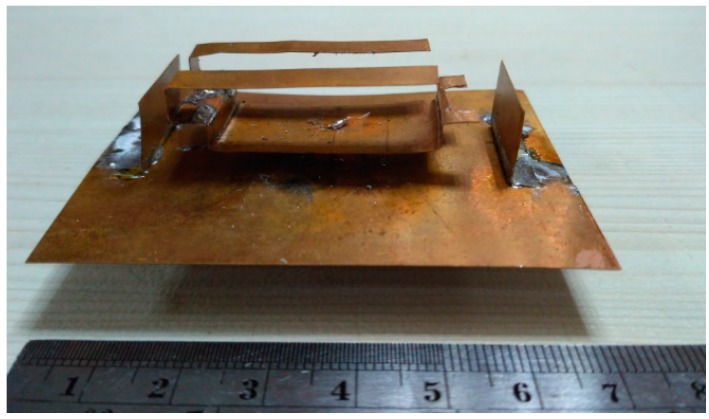
Fabricated prototype of the antenna.

A prototype of the antenna was fabricated using the parameters listed in Table 1. The fabricated antenna prototype is shown in Figure 2. The main radiating element of the antenna is taken from a 0.2 mm thick copper plate and resized accordingly. Lastly, it has been folded to provide the shape illustrated in the design. The reflector walls standing to the left and right side of the main radiator are also shaped from copper sheets and they are soldered on the ground plane.

## 3. Results and Discussion

**Figure 3:**
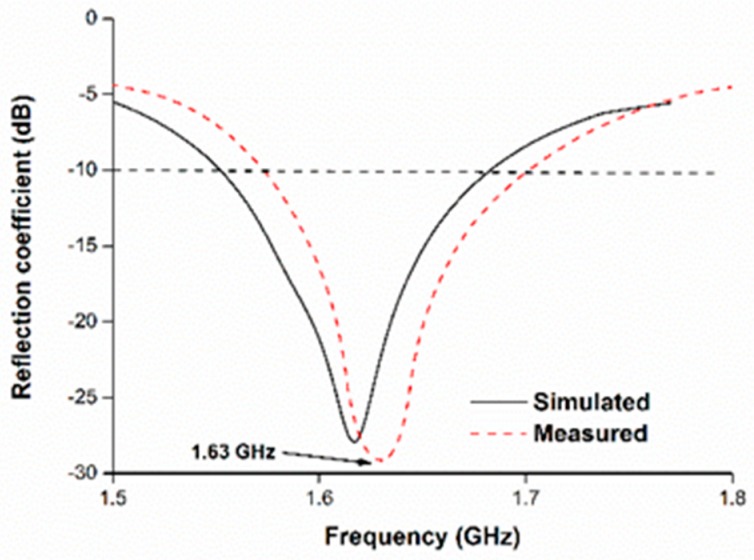
Reflection coefficients of the proposed antenna.

To validate the operating principle of the simulated design of the proposed modified PIFA antenna has been fabricated and measured. Measurement of the reflection coefficient was performed using the PNA network analyzer. Figure 3 depicts the reflection coefficient the proposed antenna. The antenna achieved its operating band at 1.55–1.68 GHz, with 130-MHz bandwidth. In the simulation, the antenna exhibited resonance at 1.61 GHz. The resonant frequency right-shifted from 1.61 to 1.63 GHz in the measurement. The operating bandwidth remained almost the same in the measurement scenario. Overall, the simulation and measurement results of the reflection coefficient are in good agreement. A slight mismatch occurred due to manual fabrication.

**Figure 4:**
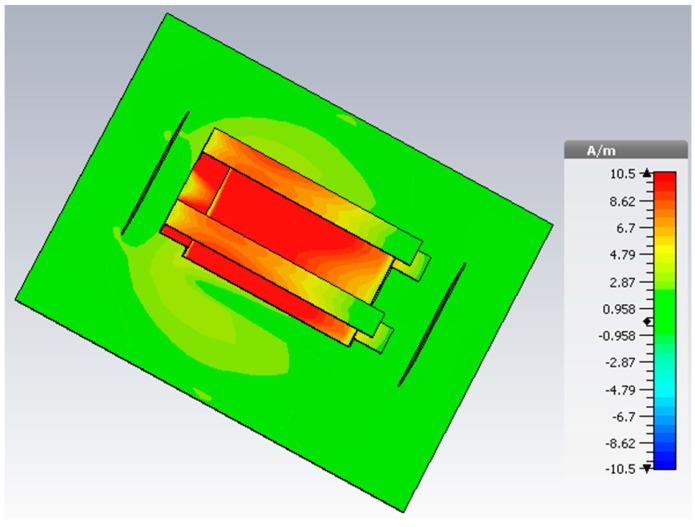
Surface current distribution of the antenna at 1.63 GHz.

The surface-current distribution of the antenna is shown in Figure 4. The operating band of the antenna is elevated by the folded structure because of the coupling between the upper radiating arms lower radiating element. A strong current can be noticed at the from lower to upper radiating element of the modified PIFA radiating structure. It plays a vital role in achieving the unidirectional radiation property. The dominant current travels from the lower radiating element to the upper radiating arms, which increases the effective radiating length and allows the antenna to resonate at a lower microwave frequency band.

**Figure 5:**
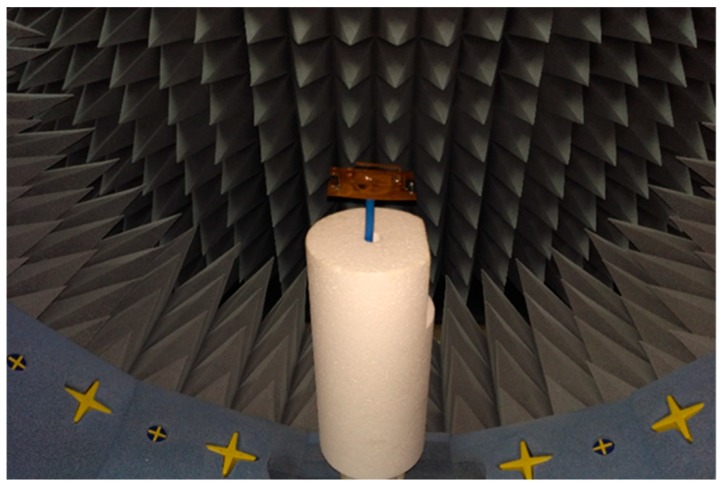
Far-field-characteristic measurement of the antenna in the Satimo near-field measurement system.

**Figure 6:**
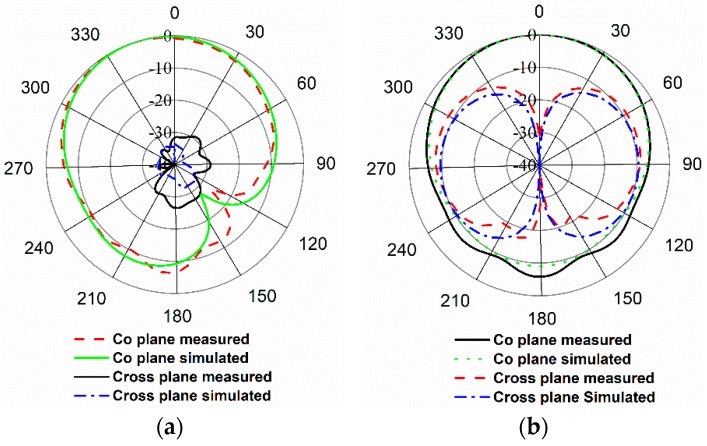
Simulated and measured radiation patterns at 1.63 GHz for (**a**) phi = 0° and (**b**) phi = 90°.

**Figure 7:**
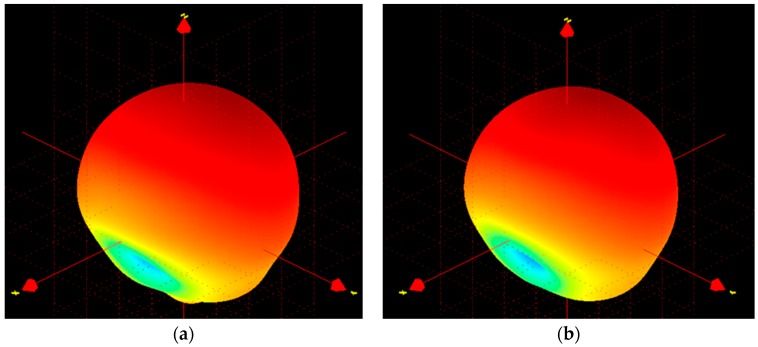
Measured 3D radiation pattern at (**a**) 1.63 GHz and (**b**) 1.68 GHz.

The far-field characteristics of the proposed antenna were achieved using the Satimo near-field measurement system, as shown in Figure 5. The far-field radiation characteristics of the antenna at phi = 0° and phi = 90° are shown in Figure 6, which clearly indicate that the antenna has a directional radiation pattern. The main lobe directivity magnitude is 4.58 dBi. The antenna obtains a 3-dB angular beam width of 130°. The results of the front-to-back ratio and surface-current distribution validate the radiation patterns shown in Figure 6. Cross polarization is negligible in the phi = 0 plane. It can be seen that there is good agreement between the measured and simulated results. Polarization is satisfactory with stable radiation pattern over the bandwidth. The radiation pattern of the antenna remains unidirectional over the whole operating band, which can be validated from the measured 3D radiation patterns shown in Figure 7.

**Figure 8:**
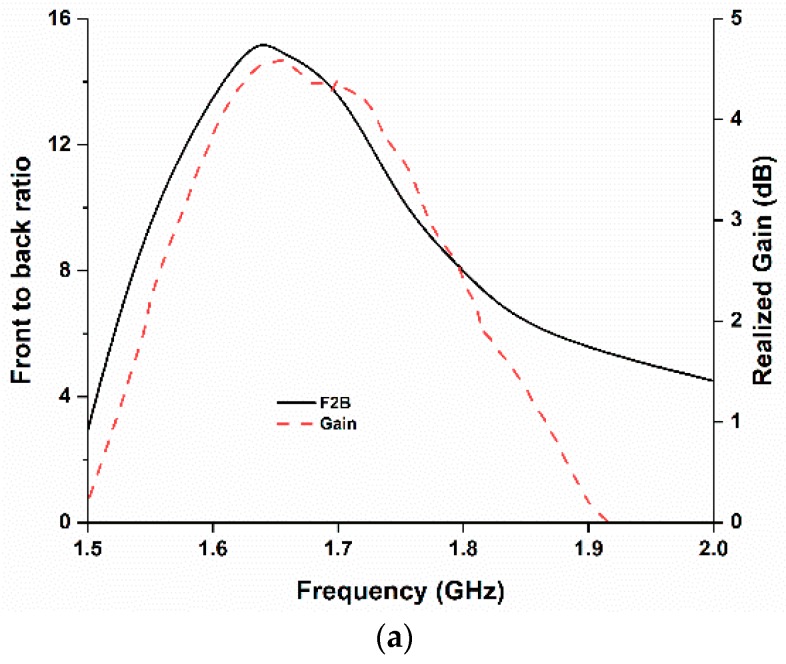

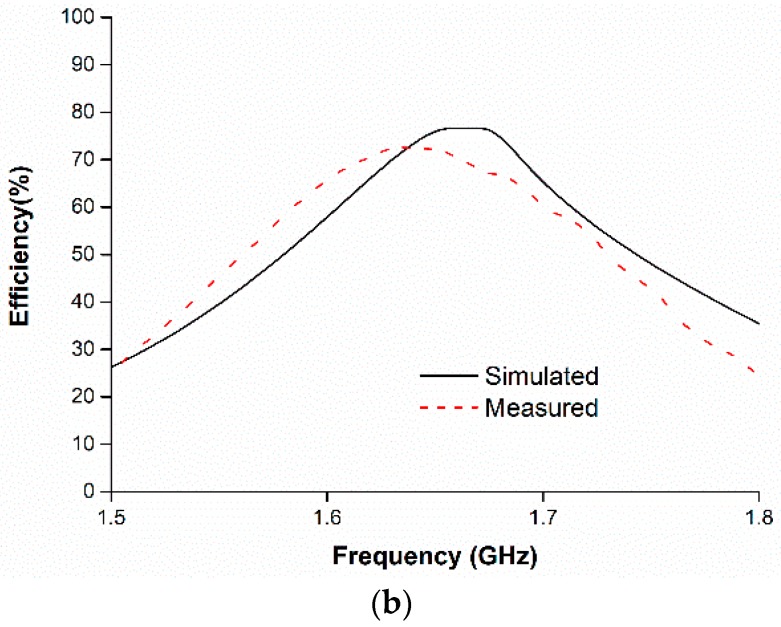
(**a**) Front-to-back ratio and realized gain (**b**) efficiency of the proposed antenna.

The current distribution remains almost the same throughout the whole operating frequency, as shown in Figure 4, which in turn maintains the existence of the directive radiation characteristics. The measured gain and front-to-back ratio are shown in Figure 8a. The antenna achieves a measured realized gain of 4.5 dB and the front-to-back ratio of 15. Moreover, the front-to-back ratio value validates the directivity radiation characteristics of the antenna. The fabricated antenna prototype achieves approximately 72% efficiency.

**Figure 9:**
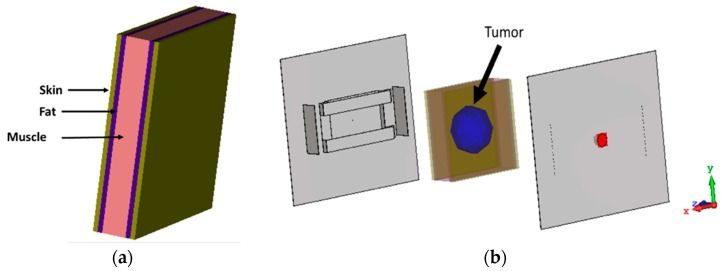
(**a**) Human tissue phantom; (**b**) simulation setup for microwave imaging.

**Figure 10:**
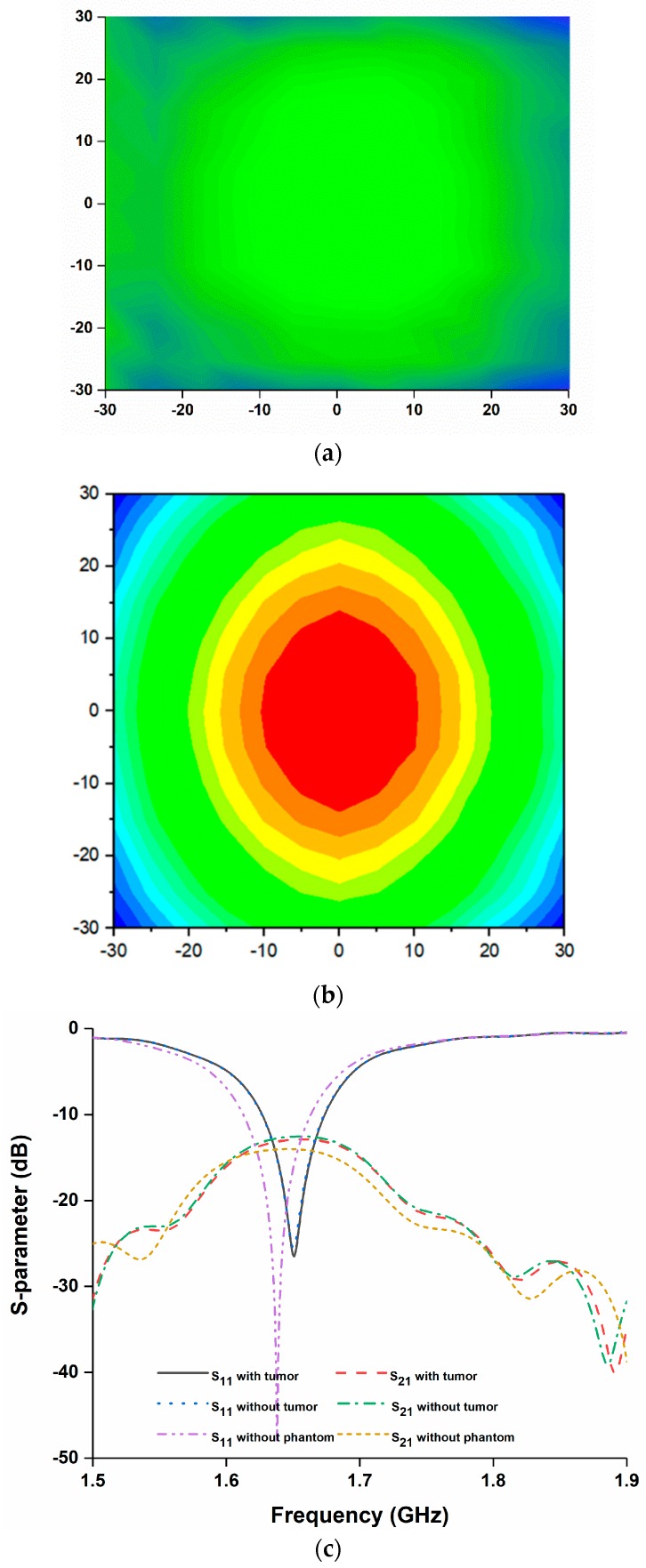
(**a**) Normalized Imaging result of human tissue without tumor; (**b**) Normalized Imaging result of human tissue with tumor; (**c**) S-parameters with and without tumor.

The microwave imaging simulation was performed using the proposed antenna as an imaging sensor. Figure 9 shows the simulation configuration of a typical microwave-imaging system to detect an abnormality in a rectangular human-tissue phantom (Figure 9a). The phantom consists of skin, fat and muscle. The dielectric constant of skin, fat and muscle are 37, 5.3 and 52 respectively. The phantom is placed between two antennas. The distance from the sensor backplane to the phantom model is 40 mm. A cyst is placed inside the tissue model, which acts as stroke or blood clot and a dielectric constant of 67 is considered and diameter is 10 mm. In this simulation, one antenna is the transmitter and the other one acts as the receiver. This study is performed to validate the performance of the proposed antenna as microwave imaging sensor. The data has been obtained using the raster scanning method [22]. Normalized scattered parameter data have been used to construct the image in Figure 10a. The existence of the tumor can easily be identified by the reddest part of the image. A transmission and reflection coefficient plot are presented in Figure 10b. The variation of S-parameters clearly depicts the sensitivity of the antenna.

**Figure 11:**
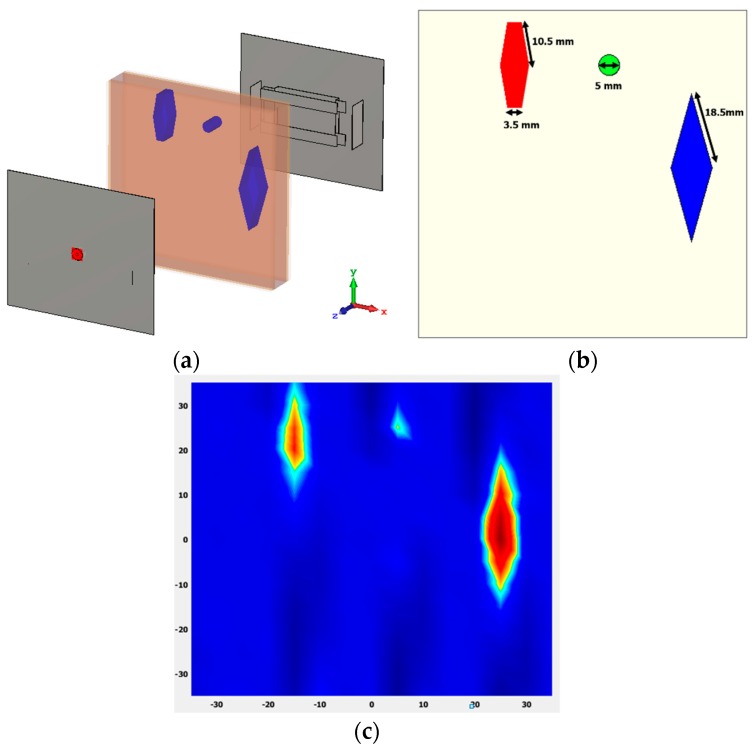
(**a**) Analysis setup; (**b**) tumor dimensions; (**c**) normalized imaging result of human tissue.

Another performance analysis has also been presented in Figure 11. Three tumors of different sizes and dielectric permittivity are placed at different positions within the human tissue. Tumor 1, tumor 2 and tumor 3 have dielectric constants of 50, 40 and 70, respectively. From Figure 11c it can be seen that three different sized tumors with different value of dielectric constant has been identified in the normalized image. Tumor 1 and 3 are clearly identified. But, tumor 2 is a little blurry because of its tiny size of 5 mm, as the step width of the scan was 2.5 mm.

**Figure 12:**
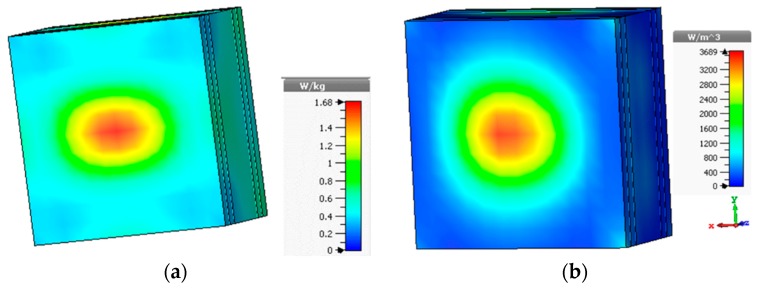
(**a**) Specific absorption rate (SAR) analysis at 1.64 GHz (10-g SAR); (**b**) power loss density over the phantom at 1.64 GHz.

The specific absorption rate (SAR) analysis was performed to observe the electromagnetic wave absorption on the phantom, as shown in Figure 12a, where the simulated power of 0.5 W was considered. It can be seen that the maximum 10 g SAR value of 1.68 W/Kg was experienced, which complies with the IEEE and ICNIRP guidelines. The power density over the human tissue phantom was analyzed, as shown in Figure 12b. We can see that a large amount of power is absorbed on the tumor concealed area due to the high dielectric property of the tumor tissue. In Figure 11b, the round and most red area shows the presence of tumor and the power density inside the phantom appears more similar to the shape of the considered tumor in the simulation.

Finally, a microwave imaging experiment was performed using the proposed sensor antenna. Two realistic breast phantoms have been used in the experiment. One of the breast phantom contains a tumor of dielectric permittivity 67. The other phantom is free from abnormality. The experiment was conducted to verify the antenna’s potential to detect the abnormality in the tumor contained breast phantom. Table 1 illustrates the important specifications of the breast phantom.

**Figure 13:**
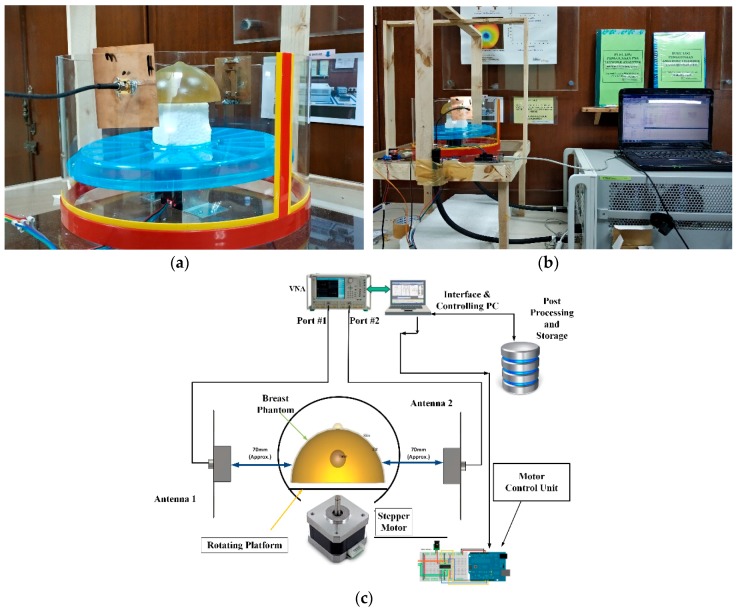
(**a**) Breast phantom placed between two sensor antennas; (**b**) experiment setup; (**c**) block diagram of the experimental setup.

**Figure 14:**
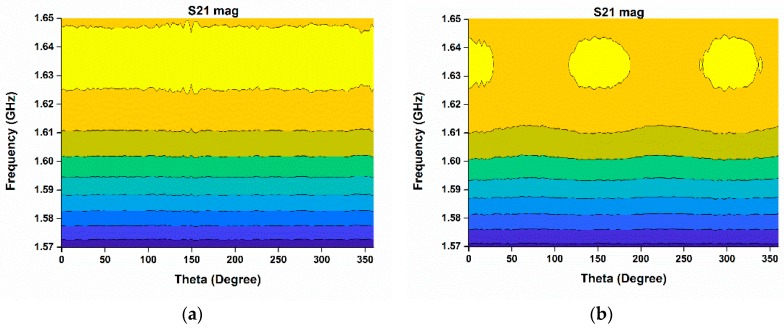
Microwave imaging result using the proposed sensor antenna; (**a**) without tumor; (**b**) with tumor.

The experimental setup has been presented in Figure 13. The experiment setup consists of two sensor antennae, the breast phantom, a motor control unit, vector network analyzer and a computer. The computer is interfaced with a vector network analyzer to collect scattered parameters of the antennas. Two antenna sensors are connected with two different ports of the VNA. The breast phantom is placed on a tray that can be rotated by a stepper motor, placed in between two sensor antennas. As the breast phantom rotates, the computer collects the scattered parameters from the vector network analyzer. For further understanding, Figure 13c depicts the block diagram of the experimental setup. After post processing the data, the imaging results of the breast phantoms are presented in Figure 14.

In Figure 14, the internal structure of the phantom breast is depicted where scattering of the electromagnetic wave is highlighted. Figure 14b clearly confirms the presence of abnormality when compared with Figure 14a. Figure 14a,b represents the experimental results of breast phantom without tumor and with tumor respectively. In Figure 14a, the electromagnetic signal left no significant variation but rather passed right through the healthy breast phantom. But, significant variations can be observed in Figure 14b, which assures the existence of a tumor or abnormality in the second breast phantom.

The proposed antenna is compared with other antennas in Table 2. Comparing the proposed antenna with others, it is observed that this antenna is an eligible candidate as a microwave imaging sensor in terms of compact size, lower frequency operation, high gain and unidirectional radiation, which are highly desired specifications.

## 4. Conclusions

The performance analysis of a modified stacked type PIFA antenna as a potential microwave imaging sensor has been discussed in this paper. Performance of the proposed sensor antenna remains consistent over the whole bandwidth with up to 72% of measured radiation efficiency. The antenna exhibited a front-to-back ratio value of 15, a unidirectional radiation property with peak realized gain of 4.5 dB and an operating frequency in the lower microwave band. The folding or stacking technique of the radiating patch aided the antenna’s good effective electrical length to achieve resonance in the lower microwave band. A computational and experimental microwave imaging study also validated the potential of the proposed antenna. High gain at a lower microwave frequency, good radiation characteristics with a unidirectional radiation pattern, high radiation efficiency and the low cost of fabrication make the proposed antenna tremendously eligible to be applied as a microwave imaging sensor for biomedical diagnosis. Though the antenna achieved all the important specifications to be used as a microwave imaging sensor, it has a comparatively small bandwidth. This can be improved further by using different bandwidth enhancement techniques of microwave antennae.

## Figures and Tables

**Table 1:** Breast phantom specification.

Parameters of Breast Phantom	Value
Dimension	16 × 8 cm^2^
Diameter of the tumor	10 mm
Number of layers	4; air, skin, breast tissue and tumor
Skin permittivity	38
Skin conductivity	1.49 S/m
Skin layer thickness	2.5 mm
Maximum width of breast tissue	8.75 cm
Breast tissue permittivity	5.14
Breast tissue conductivity	0.141 S/m

**Table 2:** Comparison with other antennas.

Refs.	Antenna Type	Size (mm)	Op. Freq. (GHz)	Gain (dB)	Directivity	Remarks
[7]	Substrate based 3D	50 × 34 × 25	1.41–3.57	2.6 dB	Almost omnidirectional	Low in gain and the radiation pattern is not unidirectional which are key requirements. Moreover, Design is expensive substrate based.
[10]	FR4 substrate based folded dipole	80 × 20 × 10	1.1–2.2	4.6 dB	Almost omnidirectional	Design is complex due to integration of substrate and copper plate. Substrate is expensive. Radiation pattern is not unidirectional.
[19]	Lens-loaded Vivaldi	110.3 × 100 × 1.6	1–14	<3 dB at lower freq. (2 GHz)	Almost omnidirectional	Large in dimension. Gain is low at lower microwave frequency with omnidirectional radiation.
[20]	Vivaldi	45 × 53	2.7–7	<2 dB in lower freq.	Unidirectional	Low in gain and height of the Vivaldi type antenna could be an issue while placement in imaging system. Substrate is also expensive.
[21]	Dipole	60 × 60 × 76.38	1–4.2	Peak gain 6.2 dB at 4 GHz, gain <3 dB at lower freq. (2 GHz)	Omnidirectional	Height is too high, complex design and the radiation pattern is not unidirectional.
[23]	Patch	Not specified	2.43–2.46	Not specified	Unidirectional	Obtained unidirectional properties. Only computational results presented, measured results of the designed antenna unavailable.
Proposed	Modified PIFA	70 × 60 × 10	1.55–1.68	4.5 dB	Unidirectional	Unidirectional radiation with high gain and efficiency in low frequency for sufficient signal penetration.
